# Cartas al Editor

**DOI:** 10.7705/biomedica.6997

**Published:** 2023-03-30

Lima, 10 de noviembre de 2022

Señores

Comité Editorial

Revista *Biomédica*

Estimados señores:

Hemos leído con sumo interés la reciente publicación de su prestigiosa revista, donde encontramos el excelente artículo original de Hurtado, *et al*. ^1^, quienes “validaron la técnica RT-LAMP colorimétrica en muestras de hisopado nasofaríngeo previamente confirmadas por RT-qPCR como prueba de referencia”. En cuanto a ello, nos permitimos resaltar el aporte de los autores, mediante experiencia en un país sudamericano que mostró la precisión diagnóstica de pruebas basadas en amplificación isotérmica mediada en lazo de transcriptasa inversa (RT-LAMP, por sus siglas en inglés), para detectar ARN de coronavirus en muestras respiratorias de pacientes.

Conviene subrayar la importancia de esta investigación, ya que propone una técnica molecular estándar para detectar SARS-CoV-2 como alternativa a la reacción en cadena de la polimerasa con transcriptasa inversa en tiempo real (RT-PCR), que es el método más usado a nivel mundial en la actualidad aunque tiene limitaciones, como el tiempo requerido para obtener resultados, que puede ser de dos a tres días, considerando además el transporte de las muestras a un laboratorio con nivel 2 de bioseguridad o superior. En el contexto de una emergencia de salud pública, como la de la COVID-19, esta demora es extremadamente desventajosa; además, los métodos comerciales basados en PCR son caros y dependen de la experiencia del operador ^2^.

De ahí, que se requiere un diagnóstico rápido de la infección por el virus SARS-CoV-2 y, por ello, se han desarrollado técnicas de amplificación isotérmica para detectar ARN virales como alternativa a la RT-PCR. En su etapa de validación analítica, esta prueba ha demostrado en diferentes estudios, incluida la presente investigación, que es fácil de implementar en un laboratorio clínico de rutina; es una tecnología significativamente rápida, de fácil manejo y no requiere reactivos o instrumentos costosos; de ahí, que sea accesible, barata y factible, lo que permite implementar medidas rápidas y efectivas de vigilancia, prevención y control epidemiológico. Considerando que es una prueba molecular, cuyo método se basa en la amplificación del ácido nucleico empleando ADN polimerasa y un conjunto de cuatro cebadores que reconocen un total de seis secuencias distintas del ADN viral en condiciones isotérmicas, exhibe mayor sensibilidad y especificidad que las pruebas de RT-PCR ^3^^-^^5^.

Aunque nos permitimos comentar que revisiones sistemáticas y metaanálisis realizados en los dos últimos años, ponen en duda la calidad de la evidencia sobre la precisión diagnóstica de las pruebas RT-LAMP desarrolladas en los estudios identificados, debido al alto riesgo de sesgo, imprecisión en los resultados y aplicabilidad incierta. Se requiere, por tanto, examinar otros aspectos intervinientes, como la influencia de la prevalencia de la enfermedad sobre el valor predictivo positivo y negativo, el rol del sitio anatómico donde se recolectó la muestra en las variaciones de la sensibilidad y la especificidad, la carga viral en los afectados, la alta tasa de falsos negativos y la incrementada heterogeneidad de los estudios ^6^^-^^7^. 

Finalmente, es conveniente considerar estos aspectos, al momento de elegir métodos de referencia precisos y confiables en el diagnóstico del SARS-CoV-2, lo cual es esencial al evaluar la precisión diagnóstica de las pruebas de detección de ácidos nucleicos.

Atentamente

César Antonio Bonilla-Asalde

Escuela de Medicina Humana, Universidad Privada San Juan Bautista, Lima, Perú https://orcid.org/0000-0002-4470-1939

David Alberto Díaz Robles

Escuela de Medicina Humana, Universidad Privada San Juan Bautista, Lima, Perú https://orcid.org/0000-0002-3256-1764

Edwin César Cieza Macedo

Escuela de Medicina Humana, Universidad Privada San Juan Bautista, Lima, Perú https://orcid.org/0000-0002-8766-1412

Oriana Rivera-Lozada

Escuela de Medicina Humana, Universidad Privada San Juan Bautista, Lima, Perú https://orcid.org/0000-0002-6546-3570
