# Supplementary material for: Cartas al Editor
Source: Biomedica. 2023 Mar 30;43(1):145–9. [Article in Spanish] doi: 10.7705/biomedica.6997 (PMC10506692; doi:10.7705/biomedica.6997)
Supplement: Related texts

# Reply: Cartas al Editor

Barranquilla, 7 de diciembre de 2022

Señores

Comité Editorial

Revista *Biomédica*

Estimados señores:

Respuesta a la carta al editor enviada por los investigadores Bonilla-Asalde, Díaz-Robles, Cieza-Macedo y Rivera-Lozada, de la Escuela de Medicina Humana de la Universidad San Juan Bautista, Lima, Perú.

De antemano, agradecemos las apreciaciones y el valor científico que se le ha dado al trabajo publicado por Hurtado-Gómez, *et al*. (DOI: 10.7705/biomedica.6523), y también, por los comentarios en cuanto a la utilidad clínica de la técnica RT-LAMP, comentarios que nos permitiremos argumentar a continuación y por qué sugerir la técnica LAMP como una técnica alternativa para diagnóstico de SARS-CoV-2.

Si bien el valor predictivo positivo y el valor predictivo negativo de un test no indican la probabilidad de que los resultados obtenidos por RT-LAMP coincidan con la realidad o el estado de salud del paciente, de acuerdo con los resultados obtenidos en nuestra investigación en relación con el momento que atravesaba la enfermedad de COVID-19 en el país, tenemos que el valor predictivo positivo (VPP) para detectar SARS-CoV2 por medio de RT-LAMP empleando el set de cebadores reportado por Broughton, fue de 0,83 y, para el set reportado por Lalli, fue de 0,81, como se muestra en el cuadro 4 ^1^.

**Cuadro 4:** Validación de RT-LAMP para diagnóstico del gen N de SARS-CoV-2. Sensibilidad, especificidad; valores predictivos de muestras positivas y negativas; concordancia por conformidad mediante el índice kappa (k) con intervalo de confianza del 95 %.

Set de cebadores	Sensibilidad	Especificidad	Valor predictivo positivo	Valor predictivo	Índice negativo kappa	Po	Pe
Set de Broughton	0,97 (0,85-1,00)	0,81 (0,65-0,92)	0,83 (0,68-0,93)	0,97 (0,83-1,00)	0,778	0,88	0,49
Set de Lalli	0,96 (0,78-1,00)	0,77 (0,55-0,92)	0,81 (0,62-0,94)	0,94 (0,73-1,00)	0,73	0,86	0,50
Sin extracción de ARN	0,95 (0,74-1,00)	0,88 (0,64-0,99)	0,90 (0,68-0,99)	0,94 (0,70;-1,00)	0,83	0,91	0,50

Índice k: valores de 0 ≥0,2 mínima concordancia, 0,2 ≥0,4 escasa concordancia, 0,4 ≥0,6 moderada, 0,6 ≥0,8 buena concordancia, 0,8 ≥1 muy buena concordancia

Estos datos sugieren que el SARS-CoV-2 tenía una gran probabilidad de ser encontrado por medio de RT-LAMP en las muestras analizadas, dado que la prevalencia de COVID-19 en las fechas del estudio (junio de 2021 a enero 2022) era bastante alta, coincidente con el periodo entre el primer y el segundo pico de la pandemia en el país. Muy probablemente, estos pacientes con resultados positivos por RT-LAMP estaban realmente enfermos, como se puede inferir también del resultado de la prueba tomada como comparativo: la PCR en tiempo real. Sin embargo, el valor predictivo negativo tuvo valores más altos utilizando ambos sets de cebadores, valores que oscilaron entre 0,97 y 0,94, lo que indica que la RT-LAMP tenía una gran probabilidad de predecir si un paciente con resultado negativo para SARS-CoV-2 realmente estaba sano (cuadro 4) ^1^.

Otros trabajo han reportado previamente que a medida que aumenta la prevalencia estimada así mismo aumentan los valores predictivos positivos, pero

Los valores predictivos negativos disminuyen ^2^. Si bien nosotros no tuvimos en cuenta la prevalencia de la COVID-19 al momento de realizar esta investigación, la pandemia por SARS-CoV-2 en Colombia estaba atravesando coincidencialmente por picos altos de contagio.

En cuanto a la variación de la sensibilidad y la especificidad de RT-LAMP en relación con el rol anatómico de la muestra, nuestro estudio incluyó dos tipos de muestras: ARN que provenía de hisopados nasofaríngeos y muestras de hisopado nasofaríngeo sin previa extracción de ARN, en los cuales RT-LAMP arrojó resultados de sensibilidad muy similares (entre el 94 y el 96 %), como se muestra en el cuadro 4. Por otro lado, Bruxvoort, *et al*., reportaron que la sensibilidad de la técnica RT-LAMP a partir de diferentes tipos de muestra, tales como hisopado orofaríngeo, hisopado nasofaríngeo, y muestras de saliva y narinas, arrojan valores muy similares de sensibilidad independientemente del tipo de muestra obtenida (93,7 %, 87,7 % y 85,4 %, respectivamente), lo que sugiere que, a pesar de existir variaciones naturales en su capacidad analítica, la técnica RT-LAMP es muy sensible para detectar muestras positivas para SARS-CoV-2 ^3^.

Con relación a la carga viral de la muestra, en el presente estudio sí se tuvo en cuenta la sensibilidad de RT-LAMP en relación con el número de copias virales presentes en la muestra. Además, se evaluó el límite de detección de la técnica. Se determinó que, en muestras con valores de Ct entre 25 y 35 o número de copias entre 21,13 copias/μl y 13 copias/pl, la sensibilidad para la reacción RT-LAMP fue del 100 %, mientras que muestras con menos 13 copias/μl hacían que la técnica RT-LAMP perdiera sensibilidad ^1^.

Otro factor interviniente en la calidad y la precisión de la técnica RT- LAMP como test diagnóstico, es la alta tasa de falsos negativos. Pu, *et al*., en su revisión sistemática reportaron que, si bien la RT-LAMP tiene una especificidad alta del 99 %, la sensibilidad fue más baja, 92 %, y la tasa de falsos negativos tuvo un valor de 12 % ^2^, un porcentaje alto que le resta a la sensibilidad de la técnica. Por el contrario, en nuestro estudio el valor o tasa de falsos negativos fue baja, del 1 % (cuadro 3) ^1^.

**Cuadro 3:** Tabla de contingencia RT-LAMP Vs. RT-qPCR correspondiente a los dos sets de cebadores dirigidos contra el gen *N* del SARS-CoV-2, set de Broughton y set de Lalli, y muestras sin extracción de ARN previa

Tabla de contingencia				
Set de Broughton	RT-LAMP	RT-qPCR		
		Positivos	Negativos	Total
	Positivos	34	7	41
	Negativos	1	30	31
	Total	35	37	72
Set de Lalli	RT-LAMP	RT-qPCR		
		Positivos	Negativos	Total
	Positivos	22	5	27
	Negativos	1	17	18
	Total	23	22	45
Sin extracción de ARN	RT-LAMP	RT-qPCR		Total
	Positivos	18	2	20
	Negativos	1	15	16
	Total	19	17	36

A pesar de que los resultados falsos positivos son un problema inherente a cualquier técnica de diagnóstico molecular, en estudios previos se ha reportado que la mayoría de los pacientes con resultados falsos negativos para COVID-19 tienden a presentar manifestaciones clínicas leves, siendo bajo el número de pacientes con resultados falsos negativos que desarrollan una enfermedad crítica con complicaciones ^4^. Tal como lo reportan Takahashi, *et al*., las altas tasas de falsos negativos por RT-LAMP pueden deberse a una baja carga viral y a un inadecuado procesamiento de las muestras, sumado a un diseño inadecuado de cebadores ^5^. En nuestro estudio, no hubo una alta tasa de falsos negativos, por lo que sugerimos la utilidad diagnóstica de la técnica RT-LAMP para el monitoreo del SARS- CoV-2, incluso con muestras crudas sin previa extracción.

Cordialmente,

Lisandro Pacheco Lugo: Orcid # 0000-0002-9248-4596

Leidy Hurtado Gómez: Orcid # 0000-0001-8371-5121

Diana Díaz Ortiz: Orcid # 0000-0002-2228-3799

Katherine Escorcia Lindo: Orcid # 0000-0002-3135-6101

Laura Flórez Oviedo: Orcid # 0000-0002-8670-8493

Yesit Bello Lemus: Orcid # 0000-0003-1006-0042

Yirys Díaz Olmos: Orcid # 0000-0003-3255-6743

Elkin Navarro Quiroz: Orcid # 0000-0001-7567-6409

Leonardo Pacheco Londoño: Orcid # 0000-0002-5770-3706

Nataly Galán: Orcid # 0000-0002-2096-7900

Ronald Maestre: Orcid # 0000-0002-5858-9829

Antonio Acosta Hoyos: Orcid # 0000-0001-7443-3982

## References

[1] Hurtado L, Diaz D, Escorcia K, Florez L, Bello Y, Diaz Y (2022). Validacion clinica de la prueba RT-LAMP para el diagnostico rapido del SARS-CoV-2. Biomedica.

[2] Pu R, Liu S, Ren X, Shi D, Ba Y, Huo Y (2022). The screening value of RT-LAMP and RTPCR in the diagnosis of COVID-19: Systematic review and meta-analysis. J Virol Methods.

[3] Bruxvoort K, Tenggardjaja CF, Slezak J, Gullett JC, Broder B, Park CH (2021). Variation in SARS-CoV-2 molecular test sensitivity by specimen types in a large sample of emergency department patients. Am J Emerg Med.

[4] Promlek T, Thanunchai M, Phumisantiphong U, Hansirisathit T, Phuttanu C, Dongphooyao S (2022). Performance of colorimetric reverse transcription loop-mediated isothermal amplification as a diagnostic tool for SARS-CoV-2 infection during the fourth wave of COVID-19 in Thailand. Int J Infect Dis.

[5] Takahashi H, Ichinose N, Okada Y. (2022). False-negative rate of SARS-CoV-2 RT-PCR tests and its relationship to test timing and illness severity: A case series. IDCases.
